# Inverse association between breast cancer incidence and degree of visual impairment in Finland

**DOI:** 10.1038/sj.bjc.6690544

**Published:** 1999-07

**Authors:** P K Verkasalo, E Pukkala, R G Stevens, M Ojamo, S-L Rudanko

**Affiliations:** Department of Public Health, University of Helsinki, PO Box 41, FIN-00014, Finland; Division of Environmental Health, National Public Health Institute, P.O. Box 95, Kuopio, FIN-70701, Finland; Finnish Cancer Registry, Liisankatu 21B, Helsinki, FIN-00170, Finland; Pacific Northwest National Laboratory, P.O. Box 999, Richland, WA, 99352, USA; Finnish Register of Visual Impairment, Mäkelänkatu 50, Helsinki, FIN-00510, Finland

**Keywords:** blindness, breast neoplasms, epidemiology, medical record linkage, melatonin, vision disorders

## Abstract

A total of 10 935 women with visual impairment were identified from the Finnish Register of Visual Impairment and followed up for cancer through the Finnish Cancer Registry for years 1983–1996. Breast cancer risk decreased by degree of visual impairment (*P* for trend 0.04) which suggests a dose–response relationship between visible light and breast cancer risk. © 1999 Cancer Research Campaign


*British Journal of Cancer* (1999) **80**(9), 1459–1460
© 1999 Cancer Research Campaign
Article no. bjoc.1999.0544

### Inverse association between breast cancer incidence and degree of visual impairment in Finland

**PK Verkasalo[1,2], E Pukkala[3], RG Stevens[4], M Ojamo[5] and S-L Rudanko[5]**

[1]Department of Public Health, PO Box 41, FIN-00014 University of Helsinki, Finland; [2]National Public Health Institute, Division of Environmental Health, P.O. Box 95, FIN-70701 Kuopio, Finland; [3]Finnish Cancer Registry, Liisankatu 21B, FIN-00170 Helsinki, Finland; [4]Pacific Northwest National Laboratory, P.O. Box 999, Richland, WA 99352, USA; [5]Finnish Register of Visual Impairment, Mäkelänkatu 50, FIN-00510 Helsinki, Finland

**Summary** A total of 10 935 women with visual impairment were identified from the Finnish Register of Visual Impairment and followed up for cancer through the Finnish Cancer Registry for years 1983–1996. Breast cancer risk decreased by degree of visual impairment (*P* for trend 0.04) which suggests a dose–response relationship between visible light and breast cancer risk.

**Keywords:** blindness; breast neoplasms; epidemiology; medical record linkage; melatonin; vision disorders

In persons with intact vision, pineal secretion of melatonin is highest early in the morning and reduces rapidly following exposure to light (Brzezinski, 1997). Melatonin may possess anticarcinogenic properties (Blask et al, 1991), so that increased exposures to light-at-night may decrease nocturnal melatonin secrection and thus contribute to the observed increases in breast cancer incidence rates (Stevens, 1987). It has been reasoned that women who are profoundly blind, and therefore not susceptible to light-at-night, should have a reduced risk of breast cancer compared with those with intact vision (Hahn, 1991). Indeed, two epidemiological studies have observed reduced risks of breast cancer among blind women (Hahn, 1991; Feychting et al, 1998).

We have previously conducted a population-based cohort study investigating cancer incidence for over 20 primary sites among persons with visual impairments in Finland (Pukkala et al, 1999). The present paper explores in more depth the specific hypothesis that women with visual impairment may have decreased breast cancer risk. In contrast to our earlier paper, we have now excluded the 211 women with unknown degree of visual impairment, extended the cancer follow-up by one additional year and calculated the standardized incidence ratios for five instead of three categories of visual impairment.

### MATERIALS AND METHODS

The study cohort included all women with visual impairment on the Finnish Register of Visual Impairment ever since it was founded in 1983. Dates of death and emigration were obtained from the national population register and cancer data from the Finnish Cancer Registry. The cancer follow-up started on 1 January 1983, or on the date of first registration to the Finnish Register of Visual Impairment, if later, and ended at death, or on 31 December 1996, whichever was earlier. The numbers of observed cancers and person-years at risk were counted by age at cancer diagnosis (5-year age-groups), calendar period of observation (1983–1987; 1988–1992; 1993–1996), time since initial registration (<2; 2–9; ≥10 years) and latest degree of visual impairment with best correction (moderate; severe; profound low vision; near-total; total blindness). Further stratification by age at onset of visual impairment (<18; 18–39; 40–64; ≥65 years) was attempted. Expected numbers of cancers were calculated by multiplying the stratum-specific number of person-years by the corresponding cancer incidence in Finland. The observed numbers of cancers were divided by the expected numbers to obtain the standardized incidence ratios (SIR). The exact 95% confidence intervals (CI) were computed under the Poisson assumption.

### RESULTS

The study cohort comprised 10 935 women providing 56 000 person-years at risk in 1983–1996 (Table 1). Two per cent of study subjects were totally blind. Roughly one-fourth of all the person-years were before age 65 and one-third during the first 2 years of cancer follow-up.

For the whole cohort, the SIR was 0.96 for breast cancer and 1.16 for all other cancers combined (Table 2). When studied by degree of visual impairment, the SIRs for breast cancer were 1.05, 0.96, 0.79, 0.66 and 0.47, respectively, among women with moderate low vision, severe low vision, profound low vision,

**Table 1** Numbers of women with visual impairment in Finland and person-years at risk in 1983–1996, by degree of visual impairment

| Degree of visual impairment | Numbers | | Person-years | |
|---|---|---|---|---|
| | *n* | % | *n* | % |
| Moderate low vision | 6 440 | 59 | 32 213 | 57 |
| Severe low vision | 1 896 | 17 | 9 403 | 17 |
| Profound low vision | 1 538 | 14 | 8 104 | 14 |
| Near-total blindness | 856 | 8 | 4 848 | 9 |
| Total blindness | 205 | 2 | 1 627 | 3 |
| Total | 10 935 | 100 | 56 195 | 100 |

*Received 9 September 1998*
*Revised 14 December 1998*
*Accepted 20 January 1999*

*Correspondence to:* PK Verkasalo, ICRF Cancer Epidemiology Unit, Gibson Building, Radcliffe Infirmary, Oxford OX2 6HE, UK

**Table 2** Observed (O) numbers of cases for breast cancer and cancers of the remaining sites, standardized incidence ratios (SIR) and 95% confidence intervals (CI) in women with visual impairment in Finland in 1983–1996, by degree of visual impairment

| Degree of visual impairment | Breast cancer[a] | | | Cancer of other sites | | |
|---|---|---|---|---|---|---|
| | O | SIR | 95% CI | O | SIR | 95% CI |
| Moderate low vision | 81 | 1.05 | 0.84–1.30 | 385 | 1.10 | 0.99–1.21 |
| Severe low vision | 21 | 0.96 | 0.59–1.46 | 133 | 1.34 | 1.12–1.59 |
| Profound low vision | 15 | 0.79 | 0.44–1.29 | 99 | 1.13 | 0.92–1.38 |
| Near-total blindness | 6 | 0.66 | 0.24–1.44 | 49 | 1.35 | 1.00–1.79 |
| Total blindness | 1 | 0.47 | 0.01–2.63 | 9 | 1.23 | 0.56–2.33 |
| All | 124 | 0.96 | 0.80–1.15 | 675 | 1.16 | 1.07–1.25 |

[a]Test for trend ($\chi^2$): *P*-value (one-sided): 0.036.

near-total blindness and total blindness, whereas the SIR for other cancers was slightly increased in all exposure categories. The decrease in breast cancer risk was statistically significant (*P* for trend = 0.036) (Breslow and Day, 1987). When stratification by age at onset of visual impairment was attempted, only three cases of breast cancer were observed in women whose visual impairment began before age 40.

### DISCUSSION

While the overall risk of breast cancer in Finnish women with all degrees of visual impairments was similar to that in the general population, the risk of other cancers was 16% higher than expected. Breast cancer decreased constantly by degree of visual impairment, suggesting a dose–response to light, whereas the risk for other cancers did not materially change.

Our observation of a 50% decrease in the risk of breast cancer among totally blind women is based on small numbers but is not dissimilar from the risk estimates of Hahn (1991) (relative risk 0.57, based on 18 cases) and Feychting et al (1998) (relative risk 0.82, based on 16 cases). The reduced risk of breast cancer may, however, simply reflect the effects of lifestyle or other factors with potential influence on breast cancer risk, namely age at menarche and menopause, use of oral contraceptives and hormonal replacement therapy, smoking, alcohol, physical exercise, and diet (Henderson et al, 1996). We did not, unfortunately, have data that would have enabled us to investigate this possibility further. Impressions of most of these factors among blind Finnish women would suggest an increase rather than a decrease in their breast cancer risk. A real melatonin effect is, therefore, possible.

Czeisler et al (1995) have reported that suppression of melatonin secretion by bright light in some totally sightless patients, suggesting that the visual subsystem participating in the regulation of melatonin secretion is separate from the visual pathways mediating normal vision sensations. Furthermore, one may assume that the functional states of these two systems are associated because the factors deteriorating one system appear likely to also affect the other system. If this is indeed the case, the functional state of the visual system denoted in the present study as ‘the degree of visual impairment’ would have been no more than a proxy measure for the efficacy of the visual subsystem participating in the regulation of melatonin secretion.

Presuming that melatonin has a real anticarcinogenic property, the breast-cancer-specific decrease may point to a mechanism with an intermediary role for oestrogens (Blask et al, 1991; Baldwin and Barret, 1998) in the biophysical/chemical pathway from visible light to endogenous melatonin secretion and, finally, to the development of breast cancer. It is pertinent, however, that it is not known. whether, or how, the total serum melatonin levels and biological secretion patterns change with the degree of visual impairment.

In summary, our results suggesting a reduced risk of breast cancer in visually impaired women support the original study hypothesis. More conclusive results may emerge from investigation of total melatonin secretion in relation to light exposure and of the possibility of confounding effects of breast cancer risk factors; and further epidemiological studies to obtain more precise risk estimates.

### ACKNOWLEDGEMENTS

Dr P Verkasalo was supported by the Academy of Finland grant No. 42022 and the Finnish Cancer Foundation, and Dr R Stevens by the US Department of Energy under contract DE-AC06-76RLO 1830 (LDRD project).

### REFERENCES

Baldwin WS and Barret JC (1998) Melatonin: receptor-mediated events that may affect breast and other steroid hormone dependent cancers. *Mol Carcinog* **21**: 149–155

Blask DE, Pelletier DB, Hill SM, Lemus-Wilson A, Grosso DS, Wilson ST and Wise ME (1991) Pineal melatonin inhibition of tumor promotion in the N-nitroso-N-methylurea model of mammary carcinogenesis: potential involvement of antiestrogenic mechanisms in vivo. *J Cancer Res Clin Oncol* **117**: 526–532

Breslow NE and Day NE (1987) *Statistical Methods in Cancer Research. Volume II – The Design and Analysis of Cohort Studies*, pp. 96–97. International Agency for Research on Cancer: Lyon.

Brzezinski A (1997) Mechanisms of disease: melatonin in humans. *N Engl J Med* **336**: 186–195

Czeisler CA, Shanahan TL, Klerman EB, Martens H, Brotman DJ, Emens JS, Klein T and Rizzo JF (1995) Suppression of melatonin secretion in some blind patients by exposure to bright light. *N Engl J Med* **331**: 6–55

Feychting M, Österlund B and Ahlbom A (1998) Reduced cancer incidence among the blind. *Epidemiology* **9**: 490–494

Hahn RA (1991) Profound bilateral blindness and the incidence of breast cancer. *Epidemiology* **2**: 208–210

Henderson BE, Pike MC, Bernstein L and Ross RK (1996) Breast cancer. In *Cancer Epidemiology and Prevention*, 2nd edn, Schottenfeld D and Fraumeni JFJ (eds), pp. 1022–1039. New York: Oxford University Press

Pukkala E, Verkasalo PK, Ojamo M and Rudanko S-L (1999) Visual impairment and cancer: a population-based cohort study in Finland. *Cancer Causes Control* **10**: 13–20

Stevens RG (1987) Electric power use and breast cancer: a hypothesis. *Am J Epidemiol* **125**: 556–561

*© 1999 Cancer Research Campaign*

## References

[PDF_00156] Baldwin W. S., Barrett J. C. (1998). Melatonin: receptor-mediated events that may affect breast and other steroid hormone-dependent cancers.. Mol Carcinog.

[PDF_00159] Blask D. E., Pelletier D. B., Hill S. M., Lemus-Wilson A., Grosso D. S., Wilson S. T., Wise M. E. (1991). Pineal melatonin inhibition of tumor promotion in the N-nitroso-N-methylurea model of mammary carcinogenesis: potential involvement of antiestrogenic mechanisms in vivo.. J Cancer Res Clin Oncol.

[PDF_00166] Brzezinski A. (1997). Melatonin in humans.. N Engl J Med.

[PDF_00168] Czeisler C. A., Shanahan T. L., Klerman E. B., Martens H., Brotman D. J., Emens J. S., Klein T., Rizzo J. F. (1995). Suppression of melatonin secretion in some blind patients by exposure to bright light.. N Engl J Med.

[PDF_00171] Feychting M., Osterlund B., Ahlbom A. (1998). Reduced cancer incidence among the blind.. Epidemiology.

[PDF_00173] Hahn R. A. (1991). Profound bilateral blindness and the incidence of breast cancer.. Epidemiology.

[PDF_00178] Pukkala E., Verkasalo P. K., Ojamo M., Rudanko S. L. (1999). Visual impairment and cancer: a population-based cohort study in Finland.. Cancer Causes Control.

[PDF_00181] Stevens R. G. (1987). Electric power use and breast cancer: a hypothesis.. Am J Epidemiol.

